# Pregnancy outcomes following natural conception and assisted reproduction treatment in women who received COVID-19 vaccination prior to conception: a population-based cohort study in China

**DOI:** 10.3389/fmed.2023.1250165

**Published:** 2023-10-11

**Authors:** Yulu Yang, Yujie Dong, Guojing Li, Biqi Yin, Xiong Tang, Liangfang Jia, Xueke Zhang, Wenjuan Yang, Chao Wang, Xiaoqing Peng, Ying Zhang, Yunxia Cao, Xiaofeng Xu

**Affiliations:** ^1^Department of Obstetrics and Gynecology, The First Affiliated Hospital of Anhui Medical University, Hefei, China; ^2^NHC Key Laboratory of Study on Abnormal Gametes and Reproductive Tract (Anhui Medical University), Hefei, China; ^3^Key Laboratory of Population Health Across Life Cycle (Anhui Medical University), Ministry of Education of the People’s Republic of China, Hefei, China; ^4^Department of Clinical Medicine, The First School of Clinical Medicine, Anhui Medical University, Hefei, China; ^5^Department of Obstetrics and Gynecology, The Hefei First People’s Hospital, Hefei, China; ^6^Anhui Province Key Laboratory of Reproductive Health and Genetics, Hefei, China; ^7^Anhui Provincial Engineering Research Center of Biopreservation and Artificial Organs, Hefei, China; ^8^Anhui Provincial Institute of Translational Medicine, Hefei, China

**Keywords:** COVID-19, vaccination, safety, obstetric outcomes, neonatal outcomes

## Abstract

**Introduction:**

The coronavirus disease-2019 (COVID-19) pandemic has swept across the world and continues to exert serious adverse effects on vulnerable populations, including pregnant women and neonates. The vaccines available at present were designed to prevent infection from COVID-19 strains and control viral spread. Although the incidence of pregnancy cycle outcomes are not likely to increase patients vaccinated prior to pregnancy compared with unvaccinated patients based on our knowledge of vaccination safety, there is no specific evidence to support this hypothesis. Therefore, the current study aimed to investigate the association between maternal vaccination prior to conception and pregnancy outcomes.

**Methods:**

We retrospectively analyzed 2,614 women who received prenatal care and delivered in the Obstetrical Department of The First Affiliated Hospital of Anhui Medical University between February 2022 and November 2022. Of the 1,380 eligible pregnant women, 899 women who had received preconception vaccination were assigned to a vaccine group and 481 women who were not vaccinated were control group. Of the enrolled patients, 291 women received fertility treatment (141 vaccinated women, 150 unvaccinated women). The primary outcomes were pregnancy complications (hypothyroidism, gestational diabetes mellitus, pregnancy-induced hypertension, polyhydramnios, oligohydramnios, premature rupture of membranes and postpartum hemorrhage), obstetric outcomes (preterm birth rate, cesarean section rate) and neonatal outcomes (birth-weight, body length, low-birth-weight rate, rate of congenital defects, neonatal mortality and admission to the neonatal intensive care unit).

**Results:**

There was no significant difference in the incidence of complications during pregnancy and delivery when compared between the vaccine group and control group in either univariate- or multivariate-models. The type of vaccine was not associated with the odds of adverse pregnancy outcome. Among the women with infertility treatment, the vaccinated group and the unvaccinated group had similar pregnancy outcomes.

**Conclusion:**

Women who received COVID-19 vaccination prior to conception had similar maternal and neonatal outcomes as women who were unvaccinated. Our findings indicate that COVID-19 vaccinations can be safely administered prior to pregnancy in women who are planning pregnancy or assisted reproductive treatment. During new waves of COVID-19 infection, women who are planning pregnancy should be vaccinated as soon as possible to avoid subsequent infections.

## 1. Introduction

The coronavirus disease-2019 (COVID-19) has spread rapidly worldwide and has exerted significant impact on humanity (1–3). As of the 24th of January 2023, over 664 million individuals had been infected and more than 6 million had died of this disease globally. In addition to the extremely high rates of morbidity and mortality caused by COVID-19, this disease can also exert significant impact on pregnant women and their infants. Previous studies reported that pregnant women with severe acute respiratory syndrome coronavirus 2 (SARS-CoV-2) infection have an increased risk of admission to the intensive care unit (ICU), intrauterine growth restriction (IUGR), preterm birth, and death (3–6). Therefore, it is vital that we administer safe and valid precautionary interventions for pregnant women and those planning to conceive to prevent adverse pregnancy outcomes.

Vaccination is widely recognized as the most important strategy with which to prevent infection by SARS-CoV-2 (7–10). In China, the vaccination policy had spread rapidly to all parts of China by the beginning of 2021; this led to a rapid increase in the population of vaccinated individuals. Among the general population, women who plan to conceive usually receive vaccinations prior to pregnancy instead of during pregnancy; this is due to widespread concerns relating to the adverse effects of this disease on pregnant women and their fetuses (11, 12). Till the February of 2022, the vast majority of women who planned to conceive had been fully vaccinated and the first batch of them had delivered. Prior to this, the outcomes of pregnancy cycle involving preconception vaccination could not be included in analyses due to the limited time period involved (13, 14). Consequently, although clinics were already performing vaccination prior to pregnancy, we know very little about the safety of his procedure on the outcomes of pregnancy. Furthermore, it has yet to be ascertained whether the administration of different types of vaccines can influence the long-term safety of pregnant women and their neonates.

It is important to identify the effects of vaccination on the safety and reproductive outcomes of SC women who plan to conceive naturally and ART women who plan to receive assisted reproductive technology. The association between vaccination and the outcomes of pregnancy cycles following infertility treatment was not be fully elucidated. Some previous studies reported that pregnant women who received vaccination had a lower clinical pregnancy rate after fresh embryo transfer (15, 16). However, other studies reported that the vaccination of pregnant women against COVID-19 had minimal effect on the outcomes of *in vitro* fertilization (IVF) or artificial insemination outcomes (17, 18). However, there was no study concerns about the preconception vaccination with long-term outcomes.

In this study, we analyzed pregnancy, delivery, and fetal development in a long-term cohort of women in China who had received COVID-19 vaccination prior to conception. We also investigated the pregnancy outcomes of women who received COVID-19 vaccination prior to conception and subsequently received IVF or intracytoplasmic sperm injection (ICSI) treatments to determine whether vaccination was associated with adverse effects.

## 2. Methods

### 2.1. Study population

**Figure 1:**
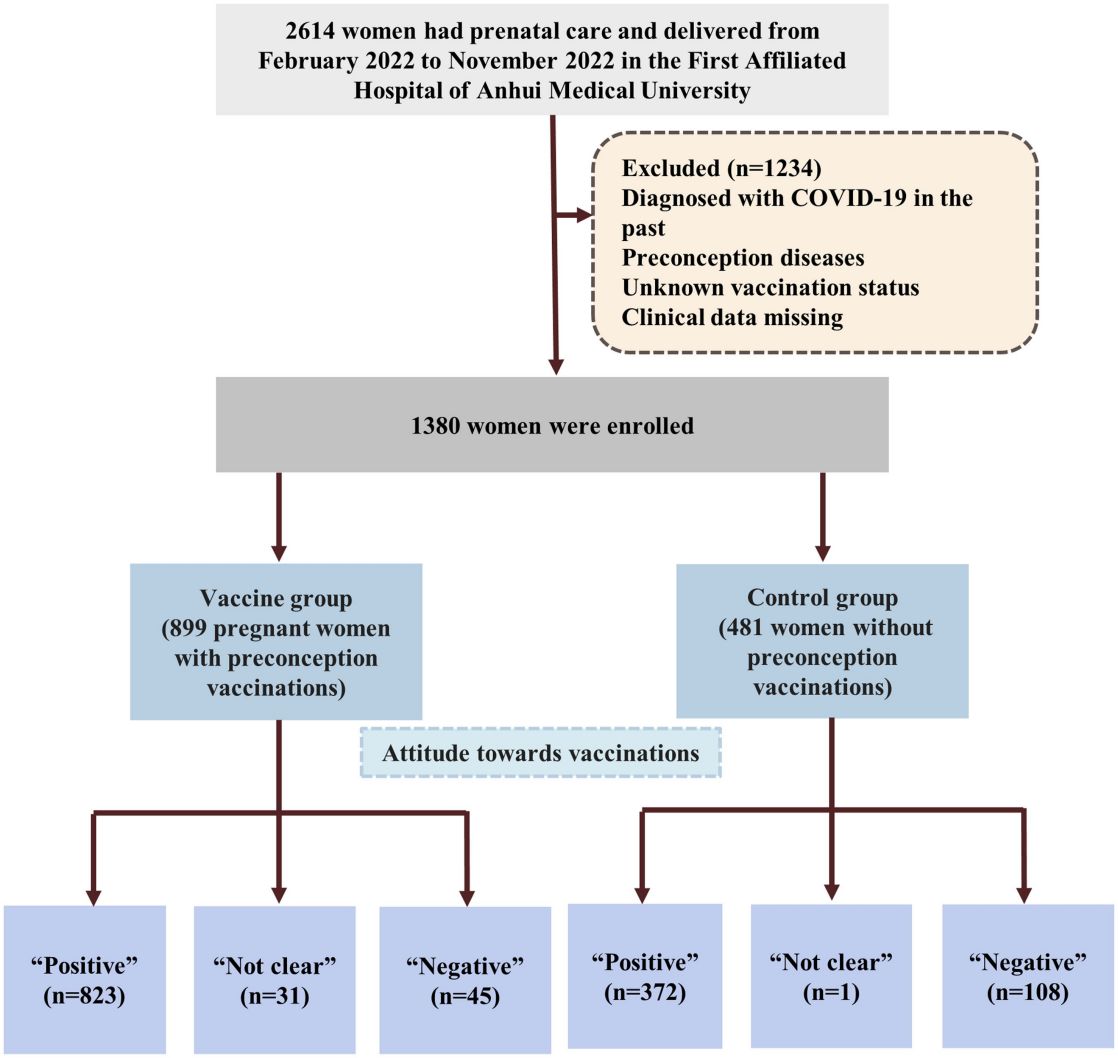
Flowchart of the women enrolled in the study. Preconception diseases including diabetes, hypothyroidism, hyperthyroidism, hyperprolactinemia, pituitary tumor, systemic lupus erythematosus, rheumatoid, rheumatic cardiopathy, sicca syndrome and mental diseases; COVID-19 vaccines are 3 types including adenovirus, inactivated and recombinant vaccines.

**Figure 2:**
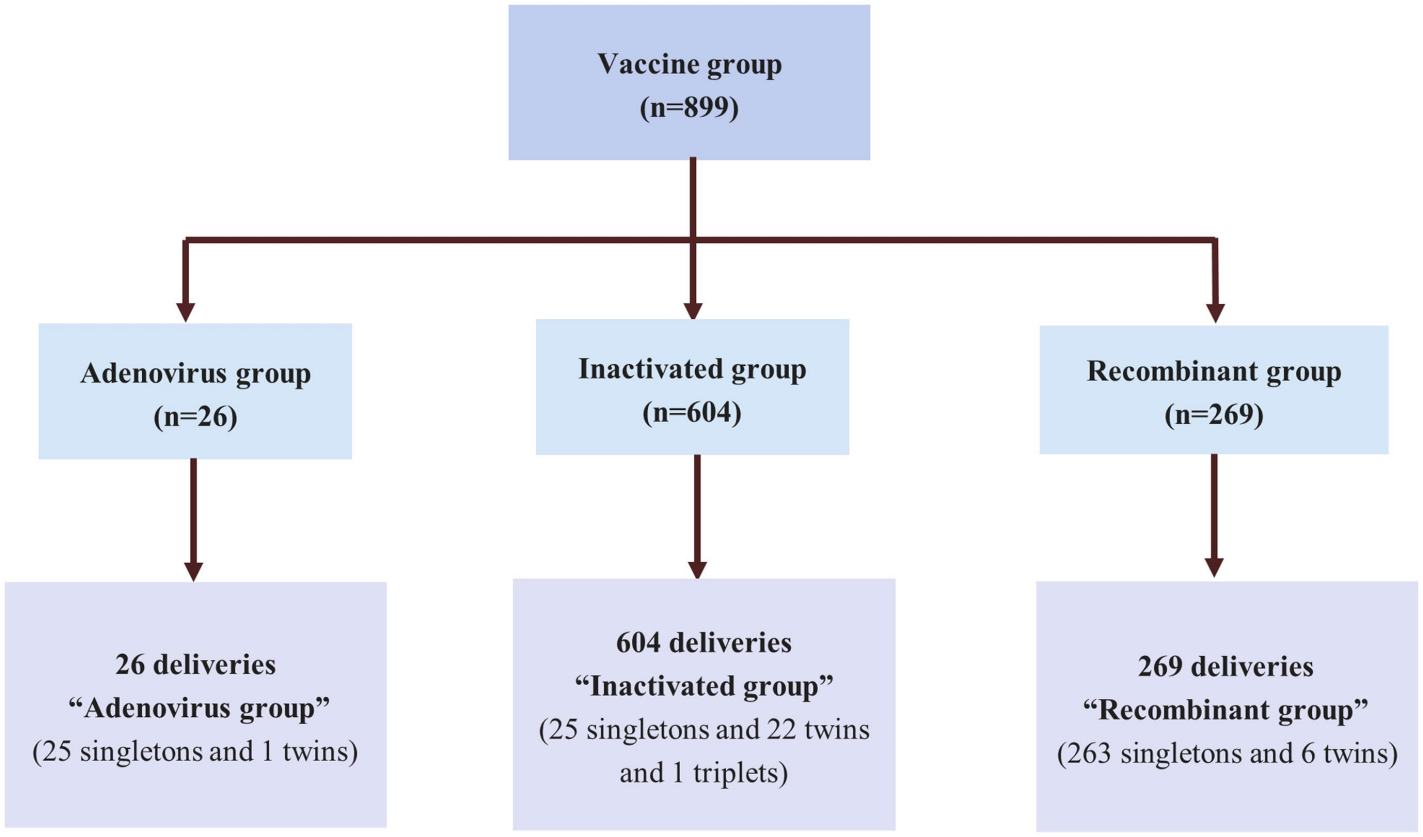
Flowchart of the women in the vaccine group.

Early in 2021, some women began to receive vaccinations prior to pregnancy. In February 2022, most women had been completely vaccinated prior to conception subsequently, some of these women became pregnant and became the first cohort of vaccinated patients to deliver. Given this background, we selected February 2022 as the start time for this population-based retrospective cohort study. Research was conducted from February 2022 to November 2022. This period represented the earliest study window for analyzing the outcomes of vaccinated pregnant women in China. During the period, 2,950 women in the Obstetrical Department of The First Affiliated Hospital of Anhui Medical University 2,614 had prenatal care and delivered. Pregnant women were included in this study if they had been inoculated or had never been inoculated with a COVID-19 vaccine during the preconception period. Pregnant women were excluded if they: (a) had been diagnosed with COVID-19 in the past; (b) preconception diseases that may have affected the outcome of pregnancy including diabetes, hypothyroidism, hyperthyroidism, hyperprolactinemia, pituitary tumor, systemic lupus erythematosus, rheumatoid, rheumatic cardiopathy, sicca syndrome and mental diseases; (c) unknown vaccination status, and (d) were missing clinical data. All eligible women were assigned to two groups: a vaccine group (adenovirus group, inactivated group, and recombinant group) and a control group (Figures 1, 2). For each pregnant women, we recorded basic, and vaccination information, adverse events during pregnancy, and delivery.

**Figure 3:**
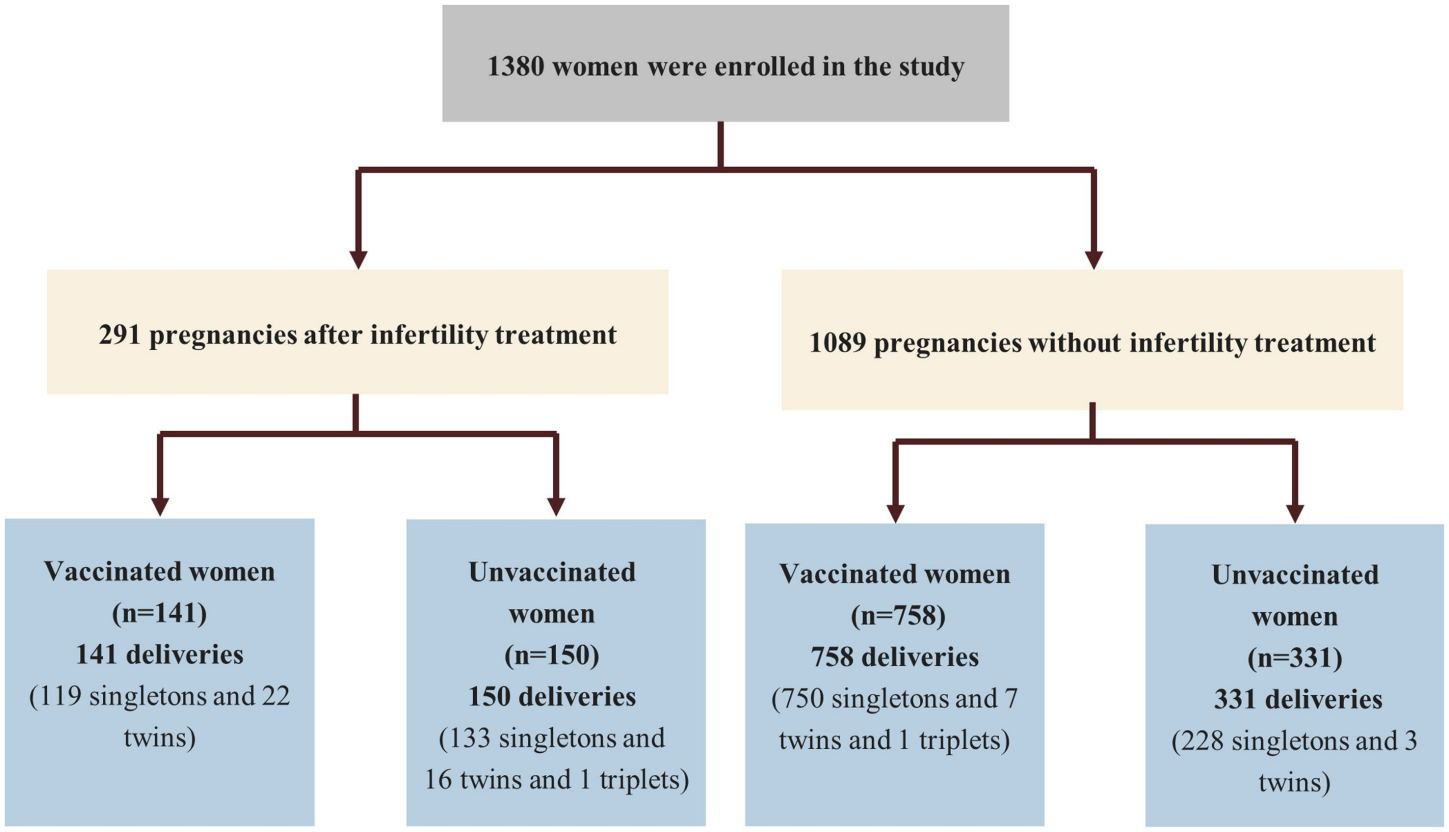
Flowchart of the women with/without infertility treatment.

Of the 1,380 women who were eligible for this study, 291 women delivered after IVF/ICSI treatments. In addition, 141 women received COVID-19 vaccinations before infertility treatment were (the vaccinated group) while 150 women who did not receive vaccination was were assigned to and unvaccinated group (Figure 3). All research procedures were carried out in accordance with the Declaration of Helsinki.

### 2.2. Vaccination and basic information

Preconception refers to the period before the last menstruation period (LMP). There are 3 types of vaccines are commonly used in China: an adenovirus, an inactivated and a recombinant vaccine. Demographic and clinical data for each pregnant women were retrieved from the Electronic Medical Records, including age, body mass index (BMI), gravidity, and the number of adverse pregnancy outcomes (APOs). Vaccination information including types of vaccines, date of vaccinations, and the patient’s attitudes towards preconception vaccination. Participants also reported whether they experienced the symptoms of fever following vaccination. All vaccination data were collected by physicians via telephone. Patients provided oral informed consent to participate in this study and the study protocol was approved by the ethics committee of Anhui Medical University (20160270).

All patients in the IVF/ICSI groups, received our center’s conventional controlled ovarian stimulation program, including the long protocol, antagonist protocol, and other protocols. We determined the dose of gonadotropin (Gn) according to the patient’s age, basic sex hormone levels, and BMI; the dose of Gn was adjusted during the follicular development. When two follicles with a diameter ≥17 mm or one follicle ≥18 mm, we administered the ovulatory dose of HCG. Oocytes were then retrieved 32–36 h later under the guidance of vaginal ultrasound. Depending on semen quality, we performed either IVF or ICSI. Normal fertilization was assessed 16–18 h after insemination/injection; then, the embryos were cultured until day 5 or day 6. Subsequently, we transferred one or two embryos after endometrial preparation. All patients received luteal support therapy; this involved dydrogesterone (10 mg, twice per day) along with progesterone intramuscular (60 mg, once per day). Clinical pregnancy was defined as a sac visible by ultrasonography with a fetal heartbeat 7 weeks after embryo transfer. A range of data retrieved from our IVF data system, age, the duration of infertility (in years), the type of infertility, the number of pregnancies, the indications of infertility, BMI, basic sex hormone levels (follicle-stimulating hormone, estradiol_2_, prolactin, luteinizing hormone, testosterone, total dosage of Gn and total days of Gn), number of retrieved oocytes, the number of transplantable embryos, the number of high-quality embryos, and the levels of sex hormone values (estradiol_2_, luteinizing hormone and progesterone) on trigger day. We also recoded endometrial thickness on the day of transfer and number of transferred embryos.

### 2.3. Indicators for evaluation

The primary indicator used for evaluation was the incidence of adverse events during pregnancy and delivery, including pregnancy complications rate (e.g., hypothyroidism, gestational diabetes mellitus, pregnancy-induced hypertension, polyhydramnios, oligohydramnios, premature rupture of membranes and postpartum hemorrhage), preterm birth rate, cesarean section rate. Hypothyroidism was defined as a serum thyroid stimulating hormone (TSH) greater than 10 mU/L (19). Gestational diabetes mellitus (GDM) was diagnosed by 75 g 2 h oral glucose tolerance test (performed between 24 and 28 weeks of gestation). The cutoffs for diagnosis at least one abnormal value in one of the following tests: a fasting glucose ≥5.1 mmol/L, 1 h ≥10.0 mmol/L, or 2 h ≥8.5 mmol/L (20). Pregnancy-induced hypertension (PIH) was defined as a systolic blood pressure (BP) ≥140 mmHg and/or diastolic BP ≥90 mmHg after 20 weeks of the pregnancy (21). Polyhydramnios was diagnosed with an amniotic fluid index (AFI) ≥25 cm while oligohydramnios was diagnosed with an AFI of ≤5 cm (22, 23). Postpartum hemorrhage was defined as blood loss >500 mL within 24 h after delivery and >1,000 mL after caesarean section (24).

The secondary indicator was neonatal outcomes included birth-weight and body length, low-birth weight (LBW) rate, congenital defects rate, neonatal mortality and neonatal intensive care unit (NICU) rate. Pregnancy outcomes for the mothers and neonates were extracted from the electronic medical records.

### 2.4. Statistical analysis

Statistical comparisons were performed between groups for baseline and clinical characteristics, vaccination information, along with obstetric and neonatal outcomes. Continuous variables were presented as mean ± standard deviation and were compared using student’s *t*-test and analysis of variance (ANOVA). Continuous data were tested for normality by visual inspection of histograms and distribution curves. Categorical variables were presented as frequencies and percentages; significance was tested with the chi-squared or Fisher’s exact test as appropriate. Univariate and multivariate regression were used to determine the association between preconception maternal COVID-19 vaccination and the outcomes of pregnancy. Model 0 was not adjusted for confounders. While, model 1 was adjusted for maternal age, BMI, gravidity, number of adverse pregnancy outcomes. Odds ratios (ORs) with 95% confidence interval (CIs) were used to compare the pregnancy outcomes between the vaccine and control groups.

Next, we compared correlations between different vaccines and pregnancy outcomes; this involved a multivariable regression model, including an interaction term related to vaccines (adenovirus, inactivated or recombinant vaccines) and a cohort variable (vaccination or non-vaccination) and other confounders. To compare associations of vaccination and pregnancy outcomes between two cohorts, we combined the two cohorts in a multivariable regression model by including an interaction term for vaccination (vaccinated or unvaccinated) with a cohort variable (SC or ART) and other confounders. In addition, multivariable regression was used to analyze the effects of vaccination in the SC/ART females. All tests were two-sided and *p*-values <0.05 indicated statistically significant differences. Data analyses were performed using SPSS (Windows version 24.0, IBM-SPSS, Chicago, IL, United States).

## 3. Results

### 3.1. Women’ demographic characteristics

One thousand three hundred eighty eligible women were enrolled and assigned to two groups. Among the participants, 899 (869 singletons, 29 twins and 1 triplet pregnancies) pregnant women who were vaccinated prior to conception were assigned to the vaccine group and 481 (461 singletons, 19 twins and 1 triplet pregnancies) pregnant women who were not vaccinated were assigned to the control group. Figure 1 shows a flowchart that summarizes the study. In the vaccine group, 26 women were inoculated with an adenovirus vaccine, 604 were inoculated with an inactivated vaccine and 269 women were received the recombinant vaccine (Figure 2). There were no significant differences between the two groups in the maternal age, BMI, gravidity and number of APOs. During our follow-up process, 823 women considered that they had not experienced negative effects on pregnancy outcomes while 45 (5.0%) women had the opposite opinion in the vaccine group. In addition, 108 (22.5%) women in the control group believed that COVID-19 vaccinations would lead to adverse pregnancy outcomes and that this was the most significant reason for their decision to remain unvaccinated. Some of them stated that they planned to receive the vaccine later or shortly after birth. Following the administration of a vaccine dose, 16 (1.8%) participants reported a fever. Of these, 9 women were inoculated with inactivated vaccine, 4 were adenovirus vaccine and 3 were recombinant vaccine. With regards to perinatal outcomes, all participants had successful births (all *p* > 0.05) (Table 1).

**Table 1:** Basic data and pregnancy outcomes of women with/without COVID-19 vaccination.

Characteristics	COVID-19 vaccination vs. control		
	Vaccine group (*n* = 899)	Control group (*n* = 481)	*p-*value
Maternal age (y)	30.71 ± 3.84	30.90 ± 3.63	0.35
BMI (kg/m^2^)	27.35 ± 3.70	27.39 ± 3.66	0.87
Gravidity (times)	1.02 ± 1.13	0.91 ± 1.17	0.10
Number of APO (times)	0.31 ± 0.67	0.37 ± 0.73	0.10
Pregnancy following fertility treatments (*n*)	141	150	0.00^*^
Attitude towards vaccination (*n*)			0.00^*^
Positive	823	372	
Not clear	31	1	
Negative	45	108	
Vaccination-induced fever (*n*)	16	—	
*Pregnancy complication*			
Hypothyroidism (*n*, %)	12.6 (113/899)	12.1 (58/481)	0.78
GDM (*n*, %)	25.0 (225/899)	26.8 (129/481)	0.47
PIH (*n*, %)	9.5 (85/899)	9.4 (45/481)	0.95
Polyhydramnios (*n*, %)	0.6 (5/899)	0.6 (3/481)	1.00
Oligohydramnios (*n*, %)	5.5 (49/899)	5.4 (26/481)	0.97
Premature rupture of membranes (*n*, %)	17.4 (156/899)	20.2 (97/481)	0.20
Postpartum hemorrhage (*n*, %)	5.6 (50/899)	7.7 (37/481)	0.12
Live birth infants (*n*)	930	502	
Preterm birth (*n*, %)	8.2 (74/899)	10.8 (52/481)	0.11
Cesarean section (*n*, %)	42.8 (385/899)	48.0 (232/481)	0.06
Birth weight (g)	3,210.92 ± 564.51	3,230.54 ± 572.62	0.53
Birth length (cm)	49.32 ± 2.93	49.16 ± 4.15	0.44
Singletons (*n*, %)	96.7 (869/899)	95.8 (461/481)	0.44
Multiples (*n*, %)	3.3 (30/899)	4.2 (20/481)	0.44
LBW rates (*n*, %)	6.9 (64/930)	8.8 (44/502)	0.20
Neonatal congenital diseases (*n*, %)	1.5 (14/930)	1.8 (9/502)	0.68
Neonatal death (*n*, %)	0 (0/930)	0 (0/502)	1.00
Other neonatal complication (*n*, %)	0.5 (5/930)	0.4 (2/502)	1.00
NICU admission rates (*n*, %)	2.6 (24/930)	3.0 (15/502)	0.65

Qualitative data are *n* (%); quantitative data are mean ± SD. BMI, body mass index; APO, adverse pregnancy outcomes; GDM, gestational diabetes mellitus; PIH, gestational hypertension; LBW, low birth weight; NICU, neonatal intensive care unit. ^*^Significant difference.

### 3.2. Pregnancy outcomes between vaccine group and control group

The incidences of hypothyroidism, gestational diabetes mellitus (GDM), pregnancy-induced hypertension (PIH), polyhydramnios, oligohydramnios, premature rupture of membranes and postpartum hemorrhage were all similar in both groups (all *p* > 0.05). Furthermore, the two groups did not differ significantly with regards to obstetric outcomes including preterm birth rate and caesarean section rate. Similarly, weight/length at birth, the incidence of LBW, neonatal congenital diseases, and NICU admission did not differ significantly between the two groups (all *p* > 0.05) (Table 1). No neonatal deaths were reported.

Association between preconception vaccination and pregnancy outcomes, as determined by unadjusted and adjusted analyses, are presented in Table 2. An adjusted analysis controlled for maternal age, BMI, number of gravidity, number of APO. No significant association was evident on multivariable regression between vaccination and the odds of preterm birth [adjusted odds ratio (aOR) 0.72, 95% CI 0.49–1.05] or with any of the following pregnancy complication: hypothyroidism (aOR 1.09, 95% CI 0.78–1.54), GDM (aOR 0.93, 95% CI 0.72–1.20), PIH (aOR 1.16, 95% CI 0.78–1.71), polyhydramnios (aOR 0.81, 95% CI 0.19–3.46), oligohydramnios (aOR 1.09, 95% CI 0.66–1.79), premature rupture of membranes (aOR 0.86, 95% CI 0.65–1.14), postpartum hemorrhage (aOR 0.70, 95% CI 0.45–1.10), or caesarean section (aOR 0.81, 95% CI 0.64–1.02), or LBW (aOR 0.74, 95% CI 0.47–1.16), or neonatal congenital diseases (aOR 0.83, 95% CI 0.36–1.91), or NICU admission (aOR 0.77, 95% CI 0.40–1.50).

**Table 2:** Association of preconception maternal COVID-19 vaccination with pregnancy outcomes on unadjusted and adjusted analysis.

Outcomes	Unadjusted OR (95% CI)	Adjusted OR (95% CI)
*Pregnancy complication*		
Hypothyroidism	1.05 (0.75–1.47)	1.09 (0.78–1.54)
GDM	0.91 (0.71–1.17)	0.93 (0.72–1.20)
PIH	1.05 (0.72–1.54)	1.16 (0.78–1.71)
Polyhydramnios	0.89 (0.21–3.75)	0.81 (0.19–3.46)
Oligohydramnios	1.01 (0.62–1.65)	1.09 (0.66–1.79)
Premature rupture of membranes	0.83 (0.63–1.10)	0.86 (0.65–1.14)
Postpartum hemorrhage	0.69 (0.45–1.08)	0.70 (0.45–1.10)
Preterm birth	0.74 (0.51–1.08)	0.72 (0.49–1.05)
Cesarean section	0.81 (0.65–1.01)	0.81 (0.64–1.02)
LBW rates	0.73 (0.47–1.13)	0.74 (0.47–1.16)
Neonatal congenital diseases	0.89 (0.39–2.05)	0.83 (0.36–1.91)
Neonatal death	1.00	1.00
Other neonatal complication	0.71 (0.16–3.20)	0.68 (0.15–3.10)
NICU admission rates	0.82 (0.42–1.58)	0.77 (0.40–1.50)

GDM, gestational diabetes mellitus; PIH, gestational hypertension; LBW, low birth weight; NICU, neonatal intensive care unit; unadjusted OR, unadjusted odds ratio under univariable; adjusted OR, adjusted odds ratio under multivariable; CI, confidence interval. Multivariable was adjusted for maternal age, BMI, number of gravidity, number of adverse pregnancy outcomes.

### 3.3. Comparison of pregnancy outcomes between the adenovirus vaccine group, inactivated vaccine group, recombinant vaccine group and controls

We divided the vaccine group into three sub-groups according to the types of vaccination received including an adenovirus vaccine group (*n* = 26), an inactivated vaccine group (*n* = 604), and an recombinant vaccine group (*n* = 269). There was no significant interaction between different types of vaccines and the vaccination in terms of pregnancy outcomes (all *p* > 0.05). Further sub-analyses were performed among those groups and control group, as shown in Supplementary Table S1. There were no significate differences between the four groups in the baseline characteristics of maternal age, BMI, gravidity and number of APO (all *p* > 0.05). The vaccination of the woman with adenovirus, inactivated, or recombinant vaccines was not associated with the pregnancy complication, odds of preterm birth, caesarean section, gestational age, LBW, new-born complications, neonatal death and NICU admission (all *p* > 0.05).

### 3.4. Basic data and pregnancy outcomes among SC/ART women

The study group consisted of 1,380 couples, including 291 couples undergoing fertility treatment. Of these, 141 women were vaccinated while 150 women were not (Figure 3). Both maternal and paternal age in the vaccinated group were significantly higher than in the unvaccinated group (*p* = 0.03 and *p* = 0.00, respectively). Basic characteristics did not different between the two groups, including BMI, gravidity, the number of APOs, duration of infertility, types of infertility, indications for infertility, basic serum total Gn dosage, total days of Gn, endometrial thickness, luteinizing hormone level, progesterone serum levels on trigger day, the number of retrieved oocytes. Moreover, the numbers of transplantable embryos, high-quality embryos, transferred embryos were similar between the vaccinated and unvaccinated groups (Supplementary Table S2).

Finally, difference in the strength of associations between vaccination and pregnancy outcomes between two cohorts was assessed by interaction analysis. There was no significant interaction between vaccination and the SC/ART cohort with regards to adverse pregnancy outcomes (all *p* > 0.05), and it indicated that association between vaccination and pregnancy outcomes was similar among SC women compare with ART women. After adjusting the maternal age, BMI, number of gravidity, number of APO in the multivariable regression model, there were no significate differences between the SC and ART cohorts (see Table 3).

**Table 3:** Pregnancy outcomes among women undergoing infertility treatment or not.

Characteristics	SC (*n* = 1,089)			ART (*n* = 291)		
	Vaccinated (*n* = 758)	Unvaccinated (*n* = 331)	OR (95% CI)	Vaccinated (*n* = 141)	Unvaccinated (*n* = 150)	OR (95% CI)
*Pregnancy complication*						
Hypothyroidism (*n*, %)	12.5 (95/758)	10.9 (36/331)	1.26 (0.83–1.91)	12.8 (18/141)	14.7 (22/150)	0.85 (0.43–1.69)
GDM (*n*, %)	24.3 (184/758)	27.2 (90/331)	0.87 (0.65–1.17)	29.1 (41/141)	26.0 (39/150)	1.05 (0.62–1.78)
PIH (*n*, %)	8.4 (64/758)	8.2 (27/331)	1.24 (0.76–2.03)	14.9 (21/141)	12.0 (18/150)	1.23 (0.61–2.48)
Polyhydramnios (*n*, %)	0.5 (4/758)	0.6 (2/331)	0.81 (0.15–4.46)	0.7 (1/141)	0.7 (1/150)	1.02 (0.06–18.54)
Oligohydramnios (*n*, %)	5.4 (41/758)	5.4 (18/331)	1.05 (0.59–1.87)	5.7 (8/141)	5.3 (8/150)	1.05 (0.38–2.95)
Premature rupture of membranes (*n*, %)	17.9 (136/758)	20.2 (67/331)	0.91 (0.65–1.27)	14.2 (20/141)	20.0 (30/150)	0.68 (0.36–1.27)
Postpartum hemorrhage (*n*, %)	4.9 (37/758)	5.1 (17/331)	0.94 (0.52–1.71)	9.2 (13/141)	13.3 (20/150)	0.61 (0.29–1.30)
Live birth infants (*n*)	767	334	—	163	168	
Preterm birth (*n*, %)	6.5 (49/758)	9.1 (30/331)	0.66 (0.41–1.07)	17.7 (25/141)	14.7 (22/150)	1.21 (0.63–2.30)
Cesarean section (*n*, %)	38.1 (288/758)	40.5 (134/331)	0.89 (0.68–1.18)	69.3 (97/141)	65.3 (98/150)	1.10 (0.66–1.85)
Birth weight (g)	3,260.67 ± 511.19	3,268.41 ± 536.17	−0.01 (−77.77 to 55.75)	3,036.78 ± 699.97	3,168.01 ± 632.91	0.04 (−186,035.76 to 416,060.07)
Birth length (cm)	49.60 ± 2.42	49.54 ± 2.61	0.01 (−0.24 to 0.40)	48.30 ± 4.27	48.97 ± 3.36	−0.04 (−1.28 to 0.66)
LBW rates (*n*, %)	4.6 (35/767)	6.3 (21/334)	0.65 (0.36–1.16)	17.8 (29/163)	13.7 (23/168)	1.37 (0.66–2.86)
Neonatal congenital diseases (*n*, %)	1.2 (9/767)	2.1 (7/334)	0.58 (0.22–1.56)	3.1 (5/163)	1.2 (2/168)	3.10 (0.56–17.23)
Neonatal death (*n*, %)	0 (0/767)	0 (0/334)	1.00	0 (0/163)	0 (0/168)	1.00
Other neonatal complication (*n*, %)	0.7 (5/767)	0.6 (2/334)	0.67 (0.14–3.21)	0 (0/163)	0 (0/168)	1.00
NICU admission rates (*n*, %)	2.3 (18/767)	3.3 (11/334)	0.64 (0.29–1.38)	3.7 (6/163)	2.4 (4/168)	1.70 (0.46–6.23)

Qualitative data are *n* (%); quantitative data are mean ± SD. GDM, gestational diabetes mellitus; PIH, gestational hypertension; LBW, low birth weight; NICU, neonatal intensive care unit.

## 4. Discussion

SARS-CoV-2 infection is known to increase the incidence of adverse pregnancy outcomes. Previous research has shown that pregnant women infected with SARS-CoV-2 have an increased risk of severe outcomes when compared with controls, including admission to the intensive care unit (ICU), PIH, preterm birth and neonatal death (25–28). Therefore, there is an urgent need to develop appropriate protocols to avoid these adverse events. Global research efforts have clearly demonstrated that vaccination is highly effective in terms of reducing the incidence of serious events (29–31). In China, the vaccination strategy began at the end of 2020 and spread significantly over the early few months of 2021. By November 2022, most of the female population planning for pregnancy had been fully vaccinated and had delivered. Due to the time period involved, women who were vaccinated before pregnancy were not included in the majority of vaccine-related clinical trials; thus, little is known about the association of vaccination and adverse pregnancy outcomes. There was no research explored the safety of preconception vaccination in women of reproductive age so far, as well as for different types of COVID-19 vaccines. Therefore, this is the first study investigating the preconception maternal COVID-19 vaccinations on pregnancy outcomes.

Vaccination in pregnancy was not associated with adverse obstetric outcomes or neonatal complications as several studies (2, 32–34). Some previous studies mentioned that COVID-19 vaccination could induce the incidence of autoimmune or inflammatory disease (28, 35–37). Almutairi et al. (38) reported that pregnant women who received vaccination may have a higher risk of adverse pregnancy complications, such as thyroid disorders. High titers of vaccine-induced antibodies like IgG against SARS-CoV-2 have been found in newborns in some studies (33, 39–42). These studies suggested that vaccine-induced antibodies were transferred across the placenta to the fetus and provides the infant with robust immunity, but further studies are needed to examine the impact of transferred immunity and their outcomes. In the present study, we found that the administration of COVID-19 vaccinations prior to conception did not increase the incidences of hypothyroidism, gestational diabetes mellitus, gestational hypertension, polyhydramnios, oligohydramnios or any of the other pregnancy complications tested. Further subgroup analyses showed that the administration of different types of vaccines prior to pregnancy had similar outcomes of pregnancy complications. Nonetheless, we will continue to increase the sample size, and continue our follow-up analyses to fully investigate the precise relationship between pre-conception vaccination and the risk of adverse pregnancy. Our research also showed that there were no significant differences in the odds of neonatal outcomes when comparing pregnant women who were vaccinated prior to conception and those who were not. The same relationship was evident for pregnant women who received different types of vaccines. Collectively, these findings indicate that pregnant women who are vaccinated prior to pregnancy are associated with similar outcomes as those who are not vaccinated.

For infertile couples, the relationship between vaccination and outcome of assisted reproductive technology remains unclear. Several studies (16, 18, 35, 43, 44) have reported that preconception COVID-19 vaccinations had no detrimental effect on either laboratory outcomes or short pregnancy outcomes after IVF/ICSI. However, Shi et al. (16) reported that vaccinated women had a significant lower rate of clinical pregnancy with fresh embryo transfer. Nevertheless, none of these previous studies investigated the association between vaccinations and long-term pregnancy outcomes after infertility treatment. In our study, we found that women who were vaccinated prior to conception had better embryo culture outcomes and higher number of transplantable embryos compared to non-vaccinated individuals. Additionally, there were no differences in obstetric and neonatal outcomes when compared between vaccinated and unvaccinated pregnant women. Collectively, these data prove that infertile couples need not delay ART treatment schedules because of the long-term safety of COVID-19 vaccinations.

Although this relatively large sample size was sufficient for a single-center investigation conducted using specific study and management protocols, the impact of COVID-19 vaccinations prior to conception on pregnancy outcomes still needs further investigation. All of the newborns in this study were healthy upon discharge. We continue to provide follow-up visits to evaluate these infants during their development.

## 5. Conclusion

Our analysis demonstrated that the COVID-19 vaccines used in China during the preconception period were not associated with adverse pregnancy outcomes, irrespective of the type of vaccine administered. Moreover, preconception vaccinations had no adverse effect on pregnant women following treatment for infertility. This study provides supportive evidence that COVID-19 vaccines can be safely used for females who are planning to become pregnant or commencing ART treatments. Furthermore, due to the risk of new waves of COVID-19 infections, women who are planning for pregnancy should be vaccinated as soon as possible.

## Data availability statement

The raw data supporting the conclusions of this article will be made available by the authors, without undue reservation.

## Ethics statement

The studies involving humans were approved by the ethics committee of Anhui Medical University. The studies were conducted in accordance with the local legislation and institutional requirements. Written informed consent for participation was not required from the participants or the participants’ legal guardians/next of kin in accordance with the national legislation and institutional requirements.

## Author contributions

YY and YD contributed to study design, data collection, analysis, and drafted manuscript. GL, BY, and XT contributed to data collection and critical revision of manuscript. LJ, XZ, WY, CW, and XP contributed to data collection and quality control. YZ, YC, and XX: project conception, study design, and critical revision of manuscript. All authors contributed to the article and approved the submitted version.
