# Supplementary material for: Pregnancy outcomes following natural conception and assisted reproduction treatment in women who received COVID-19 vaccination prior to conception: a population-based cohort study in China
Source: Front Med (Lausanne). 2023 Oct 11;10:1250165. doi: 10.3389/fmed.2023.1250165 (PMC10598612; doi:10.3389/fmed.2023.1250165)
Supplement: Supplementary file 1 [file Table_1.docx]

Supplementary Material

Pregnancy outcomes following natural conception and assisted reproduction treatment among women with preconception COVID-19 vaccination: a population-based cohort study in China

**Yulu Yang ^1,2,3†^, Yujie Dong ^1,2,3†^, Guojing Li ^1,2,3,4†^, Biqi Yin ^1,2,5^, Xiong Tang ^1,2,3^, Liangfang Jia ^4^, Xueke Zhang ^1,6,7,8^, Wenjuan Yang ^1,6,7^, Chao Wang ^1,2,3,8^, Xiaoqing Peng ^1,2,3,8^, Ying Zhang ^1,2,3*^, Yunxia Cao ^1,2,3*^ and Xiaofeng Xu ^1,2,3*^**

*** Correspondence:**Ying Zhang

ahyingjj@163.com

Yunxia Cao

caoyunxia5972@ahmu.edu

Xiaofeng Xu

xxf0550@126.com

| **Supplementary Table S1. The subgroup analysis of basic data and pregnancy outcomes of women with three types of COVID-19 vaccines or not.** | | | | | | |
| --- | --- | --- | --- | --- | --- | --- |
|  |  | **Subgroup analysis** | | | | |
| **Characteristics** |  | **Adenovirus group**  **(n=26)** | **Inactivated group**  **(n=604)** | **Recombinant group**  **(n=269)** | **Control group**  **(n=481)** | ***P* value** |
| Maternal age (y) |  | 31.50±4.27 | 30.72±3.93 | 30.60±3.59 | 30.90±3.63 | 0.53 |
| BMI (kg/m^2^) |  | 27.39±2.46 | 27.29±3.70 | 27.49±3.78 | 27.39±3.66 | 0.26 |
| Gravidity (times) |  | 1.15±1.26 | 0.99±1.15 | 1.07±1.07 | 0.91±1.17 | 0.37 |
| Number of APO (times) |  | 0.31±0.55 | 0.30±0.68 | 0.32±0.65 | 0.37±0.73 | 0.90 |
| Attitude towards vaccination (n) |  |  |  |  |  |  |
| Positive |  | 25 | 554 | 244 | 372 | - |
| Not clear |  | 0 | 21 | 10 | 1 | - |
| Negative |  | 1 | 29 | 15 | 108 | - |
| Vaccination-induced fever (n) |  | 4 | 9 | 3 | - | - |
| Pregnancy complication |  |  |  |  |  |  |
| Hypothyroidism (%, n) |  | 11.5 (3/26) | 13.2 (80/604) | 11.2 (30/269) | 12.1 (58/481) | 0.84 |
| GDM (%, n) |  | 46.2 (12/26) | 25.5 (154/604) | 21.9 (59/269) | 26.8 (129/481) | 0.06 |
| PIH (%, n) |  | 7.7 (2/26) | 9.8 (59/604) | 8.9 (24/269) | 9.4 (45/481) | 0.98 |
| Polyhydramnios (%, n) |  | 0 (0/26) | 0.8 (5/604) | 0 (0/269) | 0.6 (3/481) | 0.55 |
| Oligohydramnios (%, n) |  | 3.8 (1/26) | 5.5 (33/604) | 5.6 (15/269) | 5.4 (26/481) | 1.00 |
| Premature rupture of membranes (%, n) |  | 3.8 (1/26) | 18.5 (112/604) | 16.0 (43/269) | 20.2 (97/481) | 0.11 |
| Postpartum hemorrhage (%, n) |  | 3.8 (1/26) | 7.3 (44/604) | 1.9 (5/269) | 7.7 (37/481) | 0.41 |
| Live birth infants (n) |  | 27 | 628 | 275 | 502 |  |
| Preterm birth (%, n) |  | 3.8 (1/26) | 8.1 (49/604) | 8.9 (24/269) | 10.8 (52/481) | 0.41 |
| Cesarean section (%, n) |  | 53.8 (14/26) | 43.4 (262/604) | 40.5 (109/269) | 48.0 (231/481) | 0.15 |
| Birth weight (g) |  | 3,461.85±476.21 | 3,200.16±585.10 | 3,210.80±518.65 | 3,230.54±572.62 | 0.12 |
| Birth length (cm) |  | 49.70±2.28 | 49.33±2.99 | 49.24±2.84 | 49.16±4.15 | 0.75 |
| LBW rates (%, n) |  | 3.7 (1/27) | 7.2 (45/628) | 6.5 (18/275) | 8.8 (44/502) | 0.63 |
| Neonatal congenital diseases (%, n) |  | 3.7 (1/27) | 1.6 (10/628) | 1.1 (3/275) | 1.8 (9/502) | 0.54 |
| Neonatal death (%, n) |  | 0 (0/27) | 0 (0/628) | 0 (0/275) | 0 (0/502) | 1.00 |
| Other neonatal complication (%, n) |  | 0 (0/27) | 0.6 (4/628) | 0.3 (1/275) | 0.4 (2/502) | 0.24 |
| NICU admission rates (%, n) |  | 0 (0/27) | 2.7 (17/628) | 2.5 (7/275) | 3.0 (15/502) | 0.97 |
| Note: Qualitative data are n (%); quantitative data are mean ± SD.  *BMI*, body mass index; *APO*, adverse pregnancy outcomes; *GDM*, gestational diabetes mellitus; *PIH*, gestational hypertension; *LBW*, low birth weight; *NICU*, neonatal intensive care unit. | | | | | | |

| **Supplementary Table S2**. **Baseline demographics among women undergoing fertility treatment.** | | | | | |
| --- | --- | --- | --- | --- | --- |
|  | **Pregnancy after infertility treatment (n=291)** | |  |  |  |
| **Characteristics** | **Vaccinated group (n=141)** | **Unvaccinated group (n=150)** | ***P* value** | **t/****χ^2^** |  |
| Maternal age (y) | 32.74±3.98 | 31.73±3.67 | 0.03* | -2.26 |  |
| Paternal age (y) | 33.08±4.42 | 31.51±3.81 | 0.00* | -3.14 |  |
| BMI (kg/m^2^) | 27.79±3.20 | 27.76±3.41 | 0.95 | -0.07 |  |
| Gravidity (times) | 0.95±1.24 | 0.77±1.09 | 0.20 | -1.29 |  |
| Number of APO (times) | 0.52±1.03 | 0.41±0.71 | 0.26 | -1.13 |  |
| Duration of infertility (y) | 3.46±3.01 | 2.93±2.41 | 0.13 | -1.51 |  |
| Types of infertility (%, n) |  |  | 0.14 | 5.55 |  |
| Primary infertility | 59.6 (84/141) | 63.3 (95/150) |  |  |  |
| Secondary infertility | 40.4 (56/141) | 36.7 (55/150) |  |  |  |
| Indication of infertility (%, n) |  |  |  |  |  |
| Tubal factor | 42.6 (60/141) | 42.7 (64/150) | 0.98 | 0.00 |  |
| PCOS | 6.4 (9/141) | 12.0 (18/150) | 0.10 | \| 0.30 \| \| --- \| | |
| Male factor | 15.6 (22/141) | 18.0 (27/150) | 0.59 | 0.30 |  |
| Other | 35.4 (50/141) | 27.3 (41/150) | 0.14 | 2.23 |  |
| Basal serum sex hormone levels |  |  |  |  |  |
| E_2_ (pmol/L) | 172.80±81.38 | 164.67±79.54 | 0.46 | -0.74 |  |
| P (pmol/L) | 2.23±1.32 | 2.47±1.47 | 0.21 | 1.27 |  |
| PRL (pmol/L) | 14.64±6.14 | 15.35±6.21 | 0.40 | 0.84 |  |
| LH (pmol/L) | 5.56±3.48 | 5.18±2.79 | 0.36 | 0.92 |  |
| T (pmol/L) | 1.48±0.81 | 1.50±0.68 | 0.85 | 0.20 |  |
| Iatrogenic ovarian stimulation |  |  |  |  |  |
| Long protocol (Long term effect) | 39.0 (55/141) | 29.3 (44/150) | 0.08 | 3.03 |  |
| Antagonist protocol | 43.3 (61/141) | 51.3 (77/150) | 0.17 | 1.90 |  |
| Other | 17.7 (25/141) | 19.4 (29/150) | 0.73 | 0.12 |  |
| Total Gn dosage (U) | 2,182.70±825.23 | 2,066.57±662.89 | 0.23 | -1.21 |  |
| Total days of Gn (day) | 10.63±2.12 | 10.35±2.00 | 0.29 | -1.07 |  |
| Trigger day |  |  |  |  |  |
| Endometrial thickness (mm) | 11.10±2.74 | 11.24±3.43 | 0.91 | 0.11 |  |
| E_2_ (pmol/L) | 10,166.11±5,232.48 | 14,324.29±5,061.87 | 0.76 | 2.15 |  |
| LH (pmol/L) | 2.92±2.12 | 5.42±6.13 | 0.26 | 1.16 |  |
| P (pmol/L) | 4.69±2.03 | 5.41±2.30 | 0.49 | 0.71 |  |
| Number of retrieved oocytes | 14.45±8.62 | 14.25±7.42 | 0.84 | -1.98 |  |
| Number of transplantable embryos | 5.31±3.65 | 5.33±3.12 | 0.97 | 0.04 |  |
| Number of high-quality embryos | 5.35±4.16 | 4.52±3.44 | 0.09 | -1.72 |  |
| Number of transferred embryos | 1.40±0.49 | 1.43±0.50 | 0.54 | 0.62 |  |
| Note: Qualitative data are n (%); quantitative data are mean ± SD.  *BMI*, body mass index; *APO*, adverse pregnancy outcomes; *PCOS*, polycystic ovary syndrome; *E_2_*, estradiol_2_;  *P,* Progesterone; *PRL*, prolactin; *LH*, Luteinising hormone; *T*, Testosterone; *Gn,* Gonadotropins.  *Significant difference. | | | | | |
